# Validation of simplified uptake measures against dynamic Patlak K_i_ for quantification of lesional ^89^Zr-Immuno-PET antibody uptake

**DOI:** 10.1007/s00259-023-06151-1

**Published:** 2023-02-23

**Authors:** Jessica E. Wijngaarden, Marc C. Huisman, Yvonne W. S. Jauw, Guus A. M. S. van Dongen, Henri N. J. M. Greuter, Robert C. Schuit, Matthew Cleveland, Elske C. Gootjes, Daniëlle J. Vugts, C. Willemien Menke-van der Houven van Oordt, Ronald Boellaard

**Affiliations:** 1 Department of Radiology and Nuclear Medicine, Amsterdam UMC Location Vrije Universiteit Amsterdam, Boelelaan 1117, Amsterdam, The Netherlands; 2 Cancer Center Amsterdam, Imaging and Biomarkers, Amsterdam, The Netherlands; 3 Department of Hematology, Amsterdam UMC Location Vrije Universiteit Amsterdam, Boelelaan 1117, Amsterdam, The Netherlands; 4 Bioimaging In Vitro/In Vivo Translation (IVIVT), GlaxoSmithKline, Stevenage, UK; 5 Department of Medical Oncology, RadboudUMC, Geert Grooteplein Zuid 10, Nijmegen, The Netherlands; 6 Department of Medical Oncology, Amsterdam UMC Location Vrije Universiteit Amsterdam, Boelelaan 1117, Amsterdam, The Netherlands

**Keywords:** ^89^Zr-Immuno-PET, Quantification, Monoclonal antibody, Molecular imaging

## Abstract

**Purpose:**

Positron emission tomography imaging of zirconium-89-labelled monoclonal antibodies (^89^Zr-Immuno-PET) allows for visualisation and quantification of antibody uptake in tumours in vivo. Patlak linearization provides distribution volume (V_T_) and nett influx rate (K_i_) values, representing reversible and irreversible uptake, respectively. Standardised uptake value (SUV) and tumour-to-plasma/tumour-to-blood ratio (TPR/TBR) are often used, but their validity depends on the comparability of plasma kinetics and clearances. This study assesses the validity of SUV, TPR and TBR against Patlak K_i_ for quantifying irreversible ^89^Zr-Immuno-PET uptake in tumours.

**Methods:**

Ten patients received 37 MBq 10 mg ^89^Zr-anti-EGFR with 500 mg/m^2^ unlabelled mAbs. Five patients received two doses of 37 MBq ^89^Zr-anti-HER3: 8–24 mg for the first administration and 24 mg–30 mg/kg for the second. Seven tumours from four patients showed ^89^Zr-anti-EGFR uptake, and 18 tumours from five patients showed ^89^Zr-anti-HER3 uptake. SUV_peak,_ TPR_peak_ and TBR_peak_ values were obtained from one to six days p.i. Patlak linearization was applied to tumour time activity curves and plasma samples to obtain K_i_.

**Results:**

For ^89^Zr-anti-EGFR, there was a small variability along the linear regression line between SUV (− 0.51–0.57), TPR (− 0.06‒0.11) and TBR (− 0.13‒0.16) on day 6 versus K_i_. Similar doses of ^89^Zr-anti-HER3 showed similar variability for SUV (− 1.3‒1.0), TPR (− 1.1‒0.53) and TBR (− 1.5‒0.72) on day 5 versus K_i_. However, for the second administration of ^89^Zr-anti-HER3 with a large variability in administered mass doses, SUV showed a larger variability (− 1.4‒2.3) along the regression line with K_i_, which improved when using TPR (− 0.38–0.32) or TBR (− 0.56‒0.46).

**Conclusion:**

SUV, TPR and TBR at late time points were valid for quantifying irreversible lesional ^89^Zr-Immuno-PET uptake when constant mass doses were administered. However, for variable mass doses, only TPR and TBR provided reliable values for irreversible uptake, but not SUV, because SUV does not take patient and mass dose-specific plasma clearance into account.

**Supplementary Information:**

The online version contains supplementary material available at 10.1007/s00259-023-06151-1.

## Introduction

Positron emission tomographic (PET) imaging of 89-zirconium-labelled monoclonal antibodies (^89^Zr-mAb), known as ^89^Zr-Immuno-PET, allows for visualisation and quantification of ^89^Zr-mAb uptake in vivo. The uptake is quantified in tumours to evaluate the clinical efficacy of mAbs and in organs for toxicity evaluation purposes [1].

**Fig. 1:**
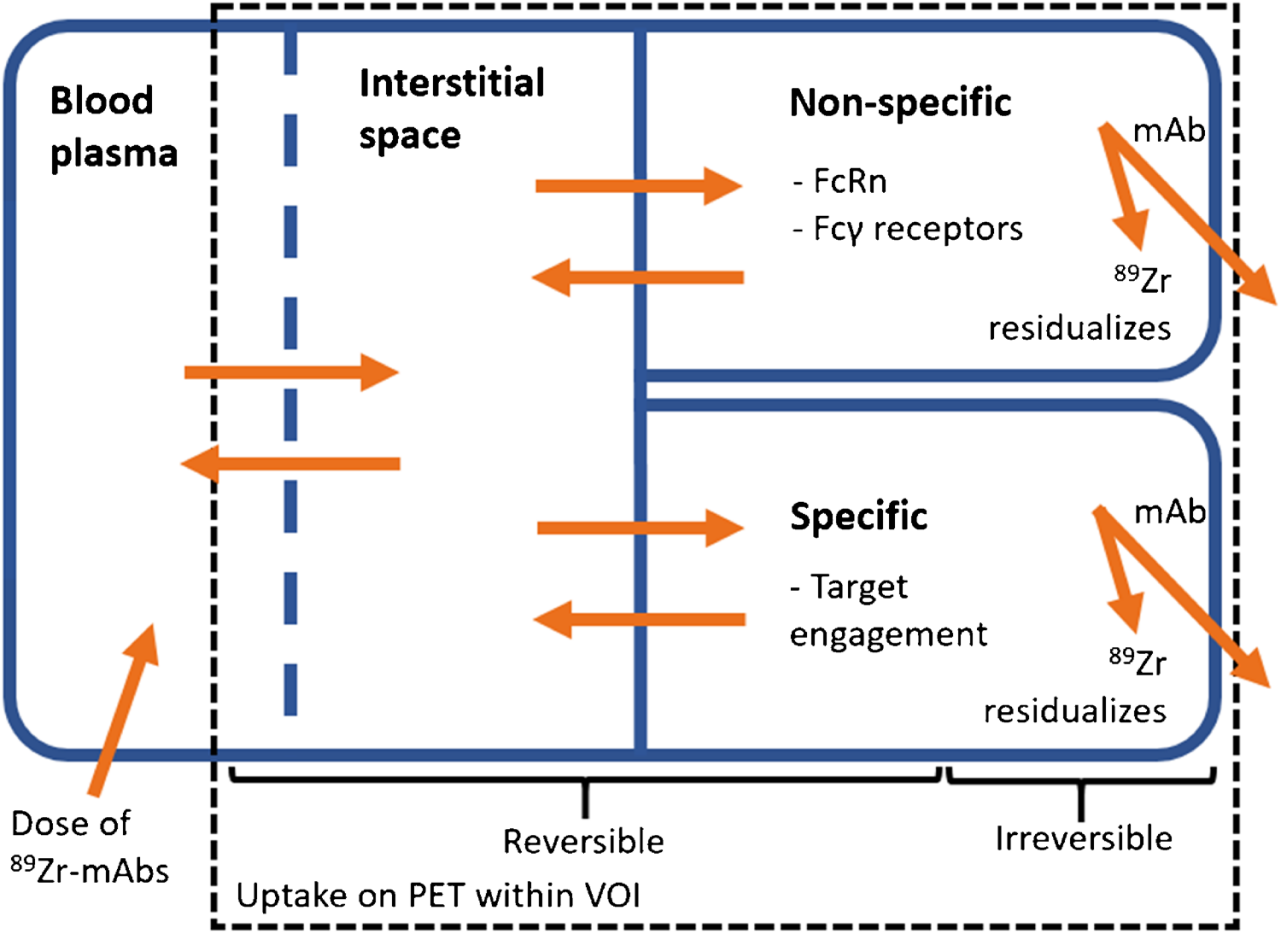
Schematic representation of the distribution and elimination of ^89^Zr-mAbs within the body. ^89^Zr-mAbs are administered to the blood plasma and distributed and are reversibly present inside the blood fraction and interstitial space of the tissue. Subsequently, several specific (binding to the target receptor) and non-specific (e.g. binding to FcRn within endothelial cells and Fcγ receptors on immunological cells) binding processes occur, which could both be reversible and irreversible. After the irreversible binding of ^89^Zr-mAb, ^89^Zr residualises inside the cell, leading to the accumulation of PET signal within the volume of interest (VOI). ^89^Zr-mAbs, zirconium-89-labelled monoclonal antibodies; FcRn, neonatal Fc-receptor; VOI, volume of interest

The measured ^89^Zr-mAb uptake results from different mechanisms of uptake. Specific target-mediated uptake in tumours and organs is of interest, but is only part of the total signal. Non-specific uptake processes also contribute to the total measured uptake. These contributions arise from ^89^Zr-mAbs being reversibly present inside the blood volume fraction and the interstitial space of the tissue [2]. Moreover, therapeutic mAbs may bind to Fcγ-receptors on immunological cells or transport via endothelial cells may occur by means of convection or receptor-mediated endocytosis [3, 4]. Within endothelial cells, mAbs that bind to the neonatal Fc-receptor (FcRn) are brought back into circulation and unbound mAbs are degraded [4]. After the degradation of ^89^Zr-mAbs, in both specific and non-specific uptake processes, the ^89^Zr stays irreversibly inside the cell, leading to the accumulation of activity measured with PET imaging [2]. In order to better interpret ^89^Zr-Immuno-PET signals, there is a need to discriminate between specific and non-specific and/or between reversible and irreversible uptake (see Fig. 1).

Patlak linearization of ^89^Zr-Immuno-PET data allows for the separation of reversible and irreversible uptake. This method is based on a two-tissue irreversible compartment model. The analysis includes the activity concentration (AC) of ^89^Zr-mAbs in tissue and in plasma on multiple days post-injection (p.i.), from which the reversible and irreversible part can be determined, providing V_T_ and K_i_ values, respectively [5]. In case specific target-mediated uptake is solely irreversible, Patlak linearization of ^89^Zr-mAb uptake brings us closer to quantifying actual target-mediated uptake. Moreover, Patlak analysis uses the measured plasma kinetics (input function) and thereby can account for differences in plasma tracer bioavailability (or clearance) between subjects, dose cohorts or imaging time points [6].

Simplified quantitative metrics, such as the standardised uptake value (SUV) and the tumour-to-plasma or tumour-to-blood ratio (TPR or TBR), are often used in PET imaging. They can be obtained from a single PET scan assessed at a certain uptake time interval. However, the correction for injected activity per patient weight as used in SUV is not necessarily a good measure for the availability of the tracer from blood to organs and lesions [7]. The validity of SUV to measure irreversible uptake depends amongst others on assumptions regarding comparability and linearity of plasma kinetics amongst subjects or conditions. Over time, the amount of administered radiolabelled tracer is physiologically cleared from the body. SUV at a certain uptake time is only valid when clearance rates are equal between patients [6]. Yet, differences in the amount of administered antibody mass (radioactively labelled and unlabelled mAbs) influence the clearance rate of ^89^Zr-mAbs [8]. TPR and TBR do take the plasma or whole blood activity concentration into account and may therefore account for differences in clearance between patients or conditions [7].

This study aims to assess the validity of SUV, TPR and TBR against Patlak K_i_ for quantifying irreversible uptake of ^89^Zr-Immuno-PET studies in tumours, exemplified with two different datasets, one using ^89^Zr-anti-EGFR, which is cetuximab given with a fixed mass dose, and ^89^Zr-labelled anti-HER3 mAb (^89^Zr-anti-HER3), which is GSK2849330 administered with variable mass doses.

## Methods

### Data overview

The current study is based on retrospective data of two ^89^Zr-immuno-PET studies. Ten patients with wild-type K-RAS colorectal cancer received 500 mg/m^2^ (range = 870–1040 mg) unlabelled mAb, followed by 37 MBq ^89^Zr-anti-EGFR (cetuximab) with 10 mg mass dose [9]. Whole-body PET/CT scans were acquired at 1–2 h, 1 day, 2 days, 3 days and 6 days p.i. in seven patients. Blood samples were drawn at every imaging time point. Data from the three patients with only late imaging time points, at 6 days and 10 days p.i., were excluded from analysis because of a missing blood sample at 24 h p.i. which affects the validity of Patlak linearization [10]. Four of the seven included patients showed ^89^Zr-mAb uptake in a total of seven tumours. Three of the seven included patients did not show tumour uptake and were excluded from the analysis. Tumours were identified on [^18^F]-FDG PET/CT at baseline, and ^89^Zr-mAb uptake was visually assessed by a nuclear medicine physician and a medical oncologist. Tumour volumes of interest (VOIs) were manually delineated on the ^89^Zr-PET scans. Protocol details including patient selection were previously published in [9].

**Fig. 2:**
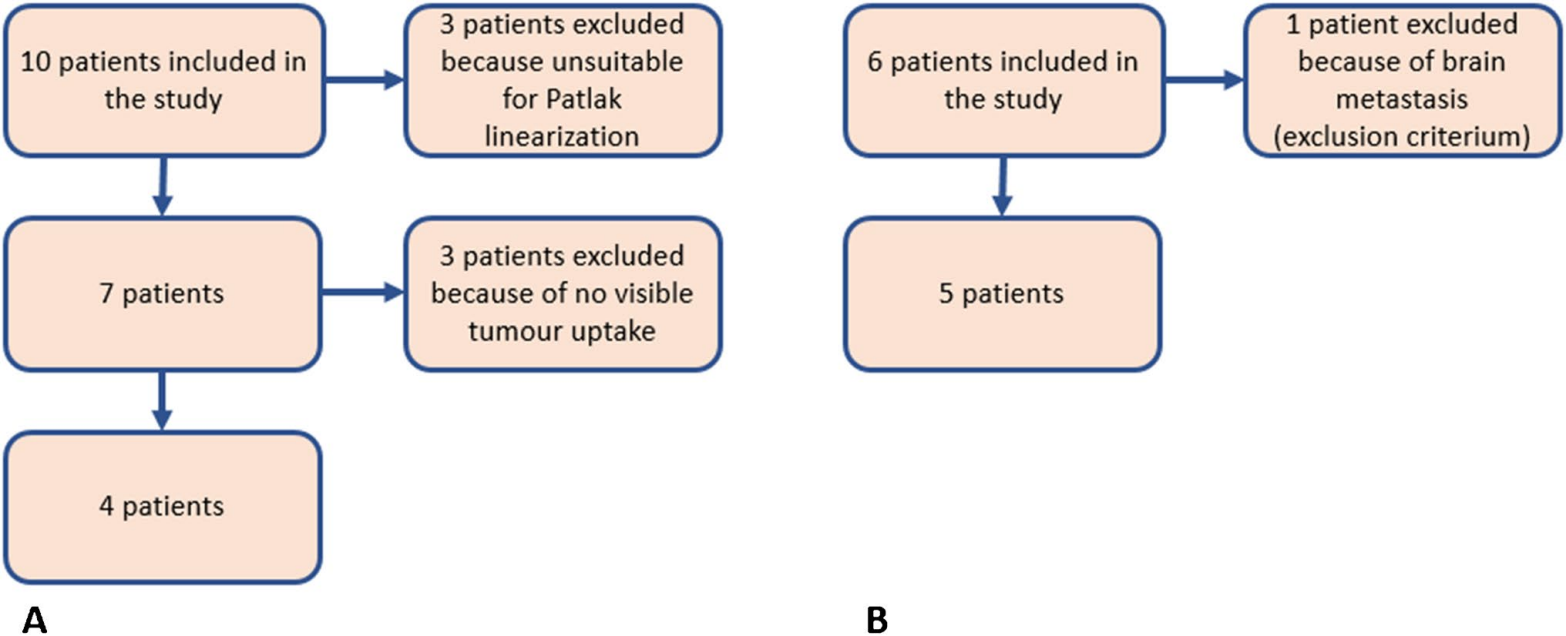
Overview of patient inclusion for the studies with ^89^Zr-anti-EGFR (**A**) and ^89^Zr-anti-HER3 (**B**)

^89^Zr-anti-HER3 mAb (GSK2849330) PET uptake data were obtained from the study presented in [8]. Six patients with HER3-positive tumours not amenable to standard treatment enrolled for the 2-part study. In part 1, they received a tracer-only dose of 37 MBq ^89^Zr-GSK2849330 with a mass dose of 8 mg or 24 mg. In part 2, 14 days later, for treatment, a variable dose of 24 mg to 30 mg/kg, unlabelled mAb was administered, followed by a dose of ^89^Zr-mAb; no other treatment was received. In both parts, whole-body PET/CT scans were acquired at 48 h and 120 h p.i. For the first three patients in part 1, an additional scan was acquired at 2 h p.i. Blood samples were drawn at 1 h, 3 h, 6 h, 12 h and 24 h p.i. and at every imaging time point. One patient was excluded from the study prior to analysis due to brain metastasis, as this was one of the exclusion criteria of the study protocol. All five remaining patients showed ^89^Zr-mAb uptake in a total of 18 tumours. ^89^Zr-mAb uptake in tumours was visually assessed by a physician with experience in PET image analysis. Tumour volumes of interest (VOIs) were manually delineated on the ^89^Zr-PET scans. Protocol details including patient selection were previously published in [8]. An overview of the patients that were included from both studies is presented in Fig. 2.

Both studies were reviewed and approved by the Central Committee on Research Involving Human Subjects of the Netherlands and the Medical Research Ethics Committee of the VU University Medical Center, the Netherlands. All patients gave written informed consent prior to study participation.

### Quantification methods

AC_peak_ values were derived from the tumour VOIs for further analysis. The SUV is defined as the activity concentration in the tumour (AC_t_), corrected for the injected activity (IA) per unit of body weight (BW) (Eq. 1) [11]. BW serves as a surrogate for the distribution volume of the injected radiolabelled tracer [6]. SUV values were obtained from each scan.

1 $$SUV= \frac{{AC}_{t}}{IA/BW}$$

The TPR or TBR [7] measures the ^89^Zr-mAb radioactivity in tumours relative to the radioactivity in blood plasma or whole blood, respectively (Eq. 2 and Eq. 3). TPR or TBR values can also be obtained by dividing the SUV or activity concentration in the tumour by the SUV or activity concentration in blood (plasma), obtained from sampling. TPR and TBR values were obtained from each scan.

2 $$TPR= \frac{{AC}_{t}}{A{C}_{p}}$$

3 $$TBR= \frac{{AC}_{t}}{A{C}_{b}}$$

Patlak linearization is based on a compartment model consisting of a reversible and an irreversible tissue compartment [5]. After the distribution of the tracer reaches equilibrium, the reversible part is proportional to the activity concentration in plasma (AC_p_) and the irreversible part is proportional to the area under the AC_p_ curve (AUC_p_). Dividing both sides by AC_p_ results in a linear relation known as the Patlak equation (Eq. 4). The slope K_i_ represents the nett influx rate of irreversible uptake [h^−1^], and the offset V_T_ is a measure for the reversible part [2, 5]. Imaging time points at 1–2 h p.i. were not included in the Patlak analyses because equilibrium between plasma and tissue compartments was not yet reached [4]. All imaging time points from 1 to 6 days p.i. were included to obtain K_i_ values.

4 $$\frac{{AC}_{t}}{A{C}_{p}}={K}_{i}\cdot \frac{A{UC}_{P}}{A{C}_{P}}+{V}_{T}$$

Two Patlak K_i_ values, one of part 1 and one of part 2 of the ^89^Zr-anti-HER3 study, were excluded from further analysis due to uncertainties in the observed data, resulting in non-plausible Patlak linearization fits, as identified by corresponding negative Patlak V_T_ values. Since the Patlak V_T_ value represents the reversible part of uptake and should be at least the blood volume fraction, this value cannot be negative [5].

### Statistical analyses

The relationship between Patlak K_i_ and SUV at different imaging time points for ^89^Zr-anti-EGFR was statistically tested using Pearson correlations. Differences between SUV, TPR or TBR values and the regression line, the residuals, were plotted against the corresponding K_i_ value on the regression line, resulting in a residuals plot. These plots give more insight into the variability between the two measures. Residuals plots were generated for SUV, TPR and TBR on day 6 p.i. for ^89^Zr-anti-EGFR and for SUV, TPR and TBR on day 5 p.i. for ^89^Zr-anti-HER3 against Patlak K_i_.

## Results

### Correlations between SUV at different imaging time points and Patlak K_i _for ^89^Zr-anti-EGFR

**Fig. 3:**
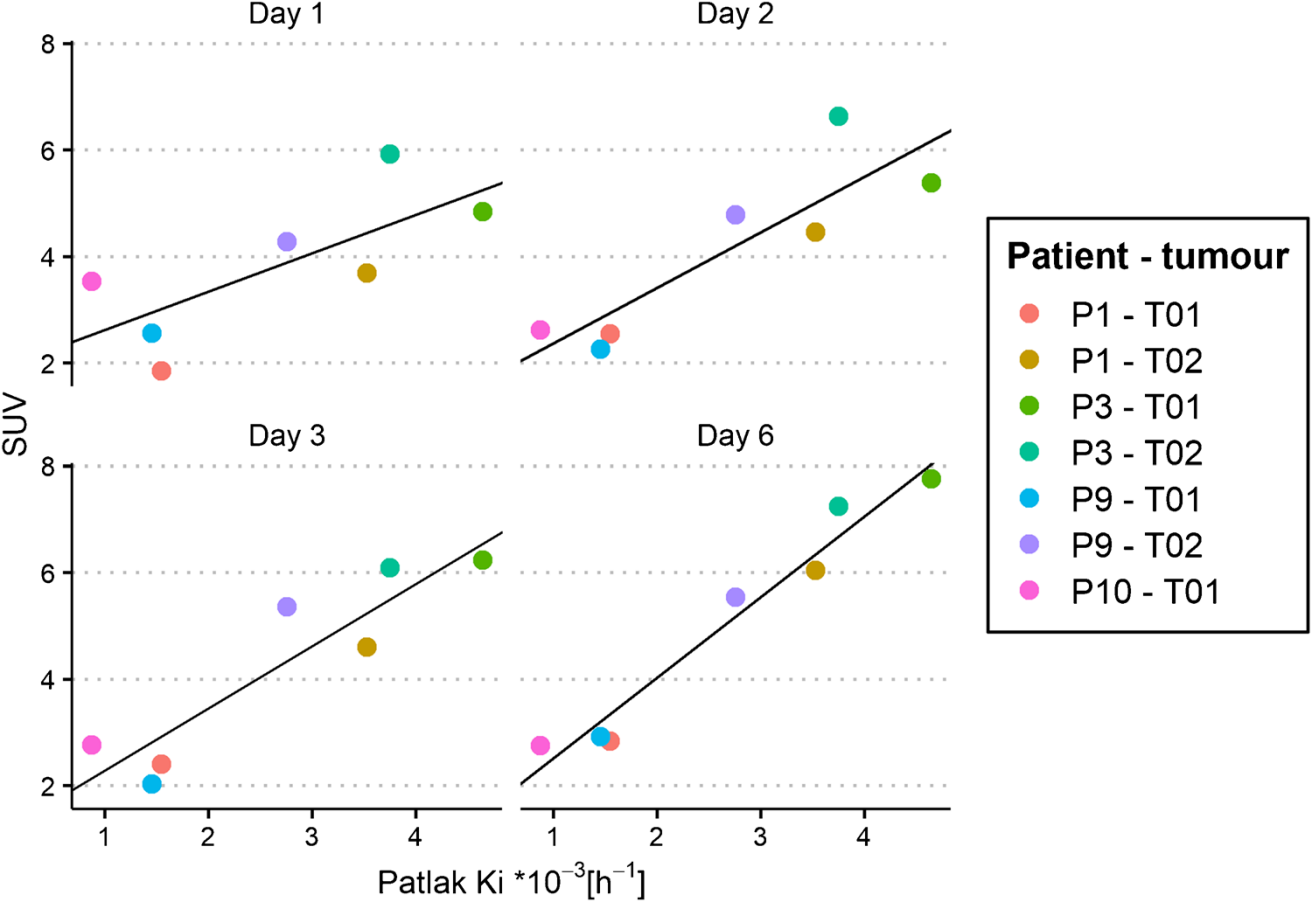
Correlations between SUV at different uptake times (days 1, 2, 3 and 6) and Patlak K_i_ for ^89^Zr-anti-EGFR uptake in seven tumours of four patients

Statistically significant positive correlations were found between Patlak K_i_ and SUV at different imaging time points (see Fig. 3). The correlation was moderate for day 1 and strong for the other three days, with an increase in strength with increasing uptake time (see Table 1). The slope of the regression line also increased with increasing uptake time.

**Table 1:** Pearson correlation coefficients and significance values for correlations between SUV at different uptake times and Patlak K_i_ for ^89^Zr-anti-EGFR uptake in seven tumours of four patients

Day	*r*	*p*-value	Slope
1	0.74	< 0.05	0.72
2	0.88	< 0.001	1.04
3	0.91	< 0.001	1.17
6	0.98	< 0.001	1.51

### Agreement between SUV, TPR and TBR on day 6 versus Patlak K_i_ for ^89^Zr-anti-EGFR

**Fig. 4:**
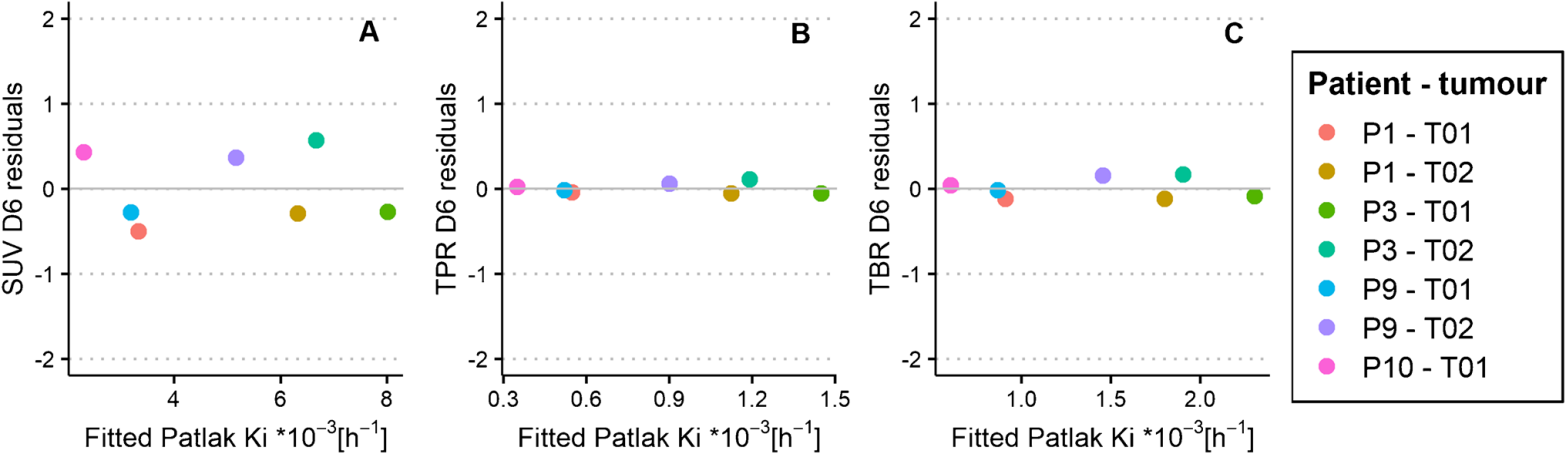
SUV (**A**), TPR (**B**) and TBR (**C**) on day 6 residuals plotted against regression-fitted Patlak K_i_ values for ^89^Zr-anti-EGFR uptake in seven tumours of four patients

Variability between SUV, TPR and TBR on day 6 versus Patlak K_i_ was visualised using residual plots (see Fig. 4). For ^89^Zr-anti-EGFR, there was a small variability along the linear regression line for SUV (− 0.51–0.57), TPR (− 0.06‒0.11) and TBR (− 0.13‒0.16) on day 6 versus Patlak K_i_. Pearson correlation plots between SUV, TPR and TBR versus Patlak K_i_ for ^89^Zr-anti-EGFR are shown in Supplementary Fig. S1.

### Dose dependency of the agreement between SUV, TPR and TBR versus Patlak K_i_ for ^89^Zr-anti-HER3

**Fig. 5:**
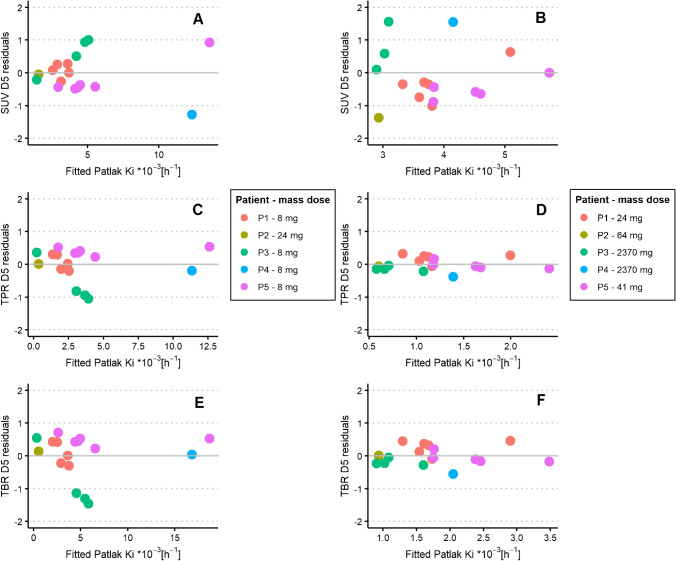
SUV, TPR and TBR on day 5 residuals plotted against regression-fitted Patlak K_i_ values for.^89^Zr-anti-HER3 uptake in 18 tumours of five patients for the first administration with similar mass doses (**A**, **C**, **E)** and the second administration with variable mass doses (**B**, **D**, **F**)

**Fig. 6:**
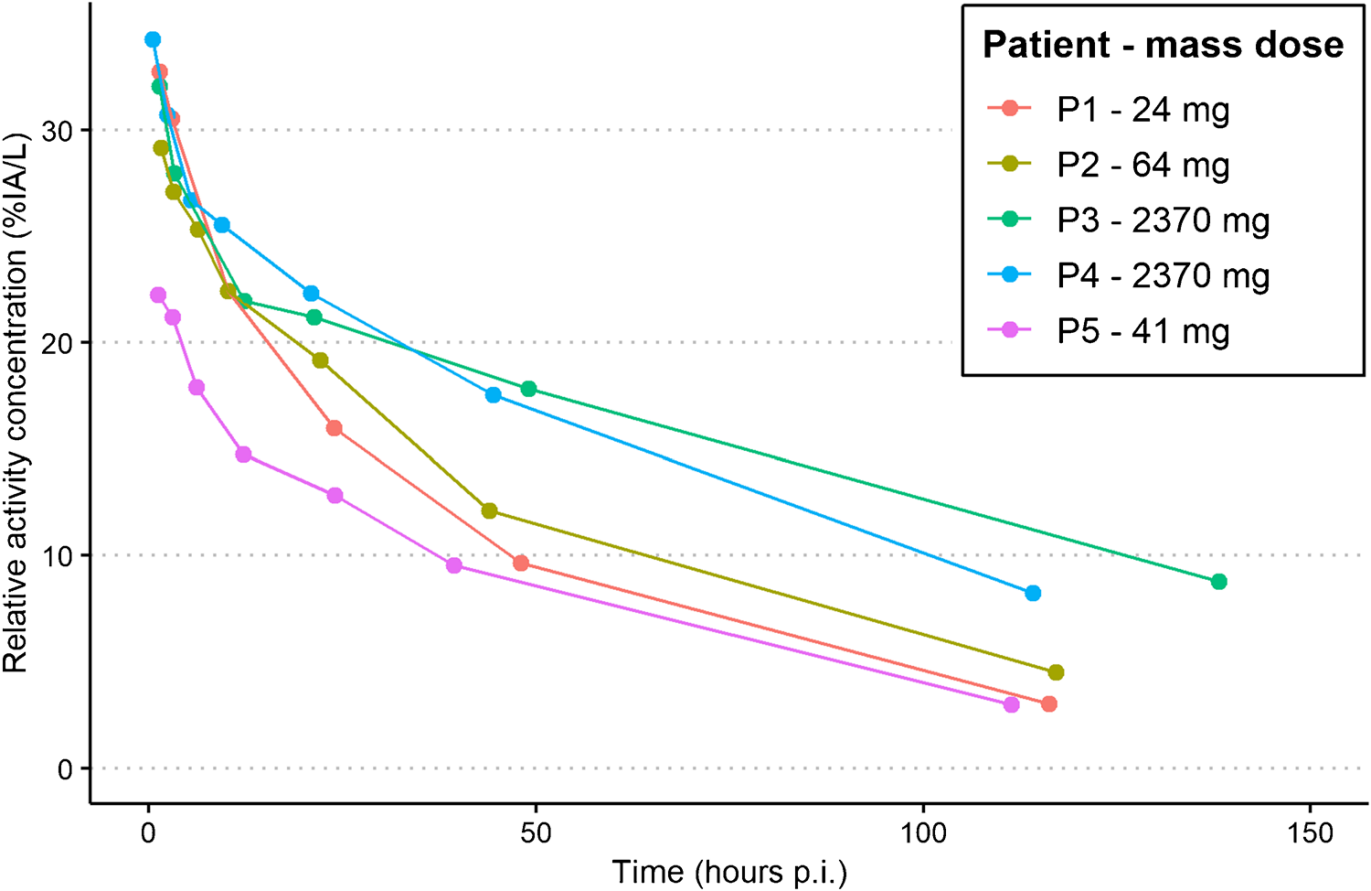
Relative activity concentration of ^89^Zr-anti-HER3 in percentage injected activity in plasma over time after the second administration with large variability in mass dose

For ^89^Zr-anti-HER3, part 1 of the study with similar administered mass doses showed similar variability for SUV (− 1.3‒1.0), TPR (− 1.1‒0.53) and TBR (− 1.5‒0.72) versus Patlak K_i_ (see Fig. 5A, C, E). Part 2 with a large variability in administered mass dose and related variability in pharmacokinetics (see Fig. 6) showed larger variability in SUV (− 1.4‒2.3) along the regression line with Patlak K_i_ (see Fig. 5B). The variability was much less for TPR (− 0.38–0.32) and TBR (− 0.56‒0.46) versus Patlak K_i_ (see Fig. 5D, F). Pearson correlation plots between SUV, TPR and TBR versus Patlak K_i_ for ^89^Zr-anti-HER3 are shown in Supplementary Fig. S2.

## Discussion

This study assessed the validity of SUV, TPR and TBR for quantification of irreversible ^89^Zr-Immuno-PET uptake in tumours. SUV at five or six days p.i. was valid for quantifying irreversible uptake of ^89^Zr-Immuno-PET in tumours when the radiotracer was given with a fixed mass dose. However, for variable mass doses, in the case of ^89^Zr-anti-HER3, TPR or TBR five days p.i. provided more reliable results than SUV due to mass dose-specific plasma clearance.

The ^89^Zr-anti-EGFR dataset contained PET imaging on multiple days (1 day, 2 days, 3 days and 6 days p.i.), providing the possibility to evaluate the validity of SUV as a function of uptake time. The SUV increased with increasing uptake time, so the SUV obtained on different imaging time points cannot be compared, which was found previously [12]. Correlations between SUV and Patlak K_i_ also differed with respect to uptake time. Firstly, longer uptake times resulted in a stronger correlation between SUV and Patlak K_i_, indicating that SUV was more reliable for measuring irreversible uptake at later time points. Secondly, the slope of the regression line increased with increasing uptake time. An increase in Patlak K_i_ results in a larger increase in SUV for later time points than for earlier time points, which indicates that SUV is more representative for irreversible uptake at later time points. SUV at the latest time point, on day 6, is therefore preferred over earlier imaging time points. Noticeably, SUV on all four imaging time points provided strong significant correlations with Patlak K_i_. The ratio between reversible and irreversible uptake is thus relatively constant between tumours, despite the uptake time.

The ^89^Zr-anti-EGFR dataset and data from the first administration of ^89^Zr-anti-HER3, both with similar mass doses within one study, showed small variability between all three simplified measures (SUV, TPR and TBR) and Patlak K_i_. The similarity between these measures means that both the contribution of irreversible relative to reversible uptake and the patient-specific plasma clearance are constant within one dataset. This possibility to use SUV for quantifying irreversible tracer uptake in tumours is favourable because SUV does not require blood sampling, can be obtained from one PET scan and is easily computable [6]. For the 8 mg dose of patient P3, TPR and TBR values showed larger variability compared to Patlak K_i_ than the other patients. Evaluation of the plasma data did not reveal discrepancies that could explain the larger variability. Nonetheless, this patient showed similar variability for SUV as for TPR or TBR.

The pharmacokinetic behaviour of these two monoclonal antibodies provides an underlying understanding of the current results. Cetuximab is primarily used in the treatment of metastatic colorectal cancer and of head and neck cancer. It blocks the epidermal growth factor receptor (EGFR), leading to tumour growth inhibition [13]. After binding to EGFR, the receptor-antibody complex is internalised and degraded, resulting in irreversible accumulation of the ^89^Zr PET signal. GSK2849330 is specific to anti-human epidermal growth factor receptor 3 (HER3), for which also internalisation and subsequent degradation are suggested [8]. This substantiates our finding that SUV, TPR and TBR show the same agreement with Patlak K_i_ values, indicating that the uptake of ^89^Zr-anti-EGFR and ^89^Zr-anti-HER3 in tumours is dominated by irreversible processes.

In contrast to data from the first administration of ^89^Zr-anti-HER3 with similar administered mass doses, the second administration with variable mass provided reliable values of irreversible uptake for TPR and TBR only, but not for SUV. In ^89^Zr-Immuno-PET studies, there is a great interest in administering varying mass doses to evaluate saturation processes. As shown by Menke van der Houven-van Oordt et al. [8], administering varying mass doses has the potential of assessing tumour target engagement, which can be utilised in optimising therapeutic dosing. As also shown in the current study, SUV is not valid for evaluating these concepts because it cannot incorporate mass dose-specific differences in uptake.

For both studies, the unlabeled mAb mass dose was administered within two hours prior to the radiolabeled dose [8, 9]. The time difference in administration is not expected to influence the pharmacokinetics since the distribution of mAbs is relatively slow. If administrated within a two-hour time frame, the pharmacokinetics of labelled and unlabeled mAbs is assumed to behave as if injected simultaneously [1]. There is a clinically practical reason for administering the unlabeled dose prior to the radiolabeled dose. After first administering the high unlabeled mass dose, clinical monitoring and intervention (if needed) would not be hampered regarding radiation safety issues because the radiolabeled is not administered yet.

A drawback of TPR, TBR and Patlak linearization is the requirement of blood sampling, which is highly patient demanding. Also, the timing for blood sampling is important; too early measurements provide reliable sampling data but not a representative AC_t_. Late uptake time measurements, however, result in very low blood activity concentrations that are less precisely measured. The latter could affect the TPR, TBR or Patlak linearization. It is important to consider these aspects when selecting blood sampling time points. An alternative for blood sampling is the assessment of the radioactivity within a blood pool region delineated on the corresponding PET scan, also known as the image-derived input function (IDIF). Previous literature has found the IDIF as a suitable surrogate for blood sampling in [^18^F-]FDG studies [14]. The IDIF contains whole blood radioactivity measurements; however, the activity concentration of plasma is of interest because ^89^Zr-mAbs available for tumour uptake are free in the blood plasma. Activity measurements in whole blood may also be valid as input, but only if the whole blood to plasma ratio is constant over time, indicating no binding of tracer to blood cells. The current study shows similar results for TPR and TBR, which implies that whole blood measurements are a valid alternative for plasma assessments. In future studies, we will evaluate if IDIF is suitable as an alternative for blood plasma samples in ^89^Zr-Immuno-PET studies.

Previous literature also showed that SUV did not provide reliable quantification of tracer uptake for several different tracers. Van den Hoff et al. [7] compared Patlak linearization to the tumour-to-blood standard uptake ratio (SUR), equal to the TPR/TBR, and to the SUV for [^18^F-]FDG uptake in patients with liver metastases of colorectal cancer. The SUR had a higher correlation with the Patlak K_i_ value than the SUV [7]. Cheebsumon et al. [15] also compared the SUV with Patlak linearization for assessing treatment response using [^18^F-]FDG PET and found that SUV may differ from full kinetic analysis results also due to changes in the plasma input function before and after treatment. Additionally, Jansen et al. [16] performed a full pharmacokinetic analysis of [^18^F]DCFPyL uptake in patients with metastasized prostate cancer. They found that SUV was not valid to quantify [^18^F]DCFPyL uptake [16].

The EORTC guidelines, established to standardise PET methodology, recommend to initially validate simplified measures, such as the SUV, to the more quantitative Patlak linearization method [6]. Results from the current study substantiate that recommendation, showing that it is applicable not only for FDG but also in the case of ^89^Zr-Immuno-PET studies. The validity of SUV for quantification of irreversible ^89^Zr-mAb uptake depended on mass dose-specific differences in plasma activity concentrations, while both TPR and TBR were valid despite differences in administered mass dose. Additionally, patient-specific differences in plasma clearance could affect the validity of SUV in ^89^Zr-Immuno-PET studies [6]. Therefore, evaluation of the correct method of quantification is essential.

## Conclusion

In conclusion, we found that SUV, TPR and TBR are valid surrogates for quantitative Patlak K_i_ in a case similar mass doses are administered. However, SUV is not valid and should not be used when the administered mass dose is varied. In general, TPR or TBR should be used for quantification of (irreversible) ^89^Zr-mAb uptake as these metrics are valid despite patient and mass dose-specific differences in plasma activity concentration.

## Supplementary Information

Below is the link to the electronic supplementary material.

Supplementary file1 (DOCX 547 KB)

## Data Availability

The data used to support the findings of this study have not been made available because they are obtained from industry sponsored clinical trials.
