# Supplementary material for: Validation of simplified uptake measures against dynamic Patlak Ki for quantification of lesional 89Zr-Immuno-PET antibody uptake
Source: Eur J Nucl Med Mol Imaging. 2023 Feb 23;50(7):1897–905. doi: 10.1007/s00259-023-06151-1 (PMC10199860; doi:10.1007/s00259-023-06151-1)
Supplement: Supplementary file 1 — Supplementary file1 (DOCX 547 KB) [file 259_2023_6151_MOESM1_ESM.docx]

Validation of simplified uptake measures against dynamic Patlak K_i_ for quantification of lesional ^89^Zr-immuno-PET antibody uptake

Jessica E. Wijngaarden^1,2^, Marc C. Huisman^1,2^, Yvonne W.S. Jauw^1,2,3^, Guus A. M. S. van Dongen^1,2^, Henri N. J. M. Greuter^1,2^, Robert C. Schuit^1,2^, Matthew Cleveland^4^, Elske C. Gootjes^5^, Daniëlle J. Vugts^1,2^, C. Willemien Menke-van der Houven van Oordt^2,6^, Ronald Boellaard^1,2^

**Author’s affiliations**^1^ Amsterdam UMC location Vrije Universiteit Amsterdam, Department of Radiology and Nuclear Medicine, Boelelaan 1117, Amsterdam, The Netherlands

^2^ Cancer Center Amsterdam, Imaging and Biomarkers, Amsterdam, The Netherlands

^3^ Amsterdam UMC location Vrije Universiteit Amsterdam, Department of Hematology, Boelelaan 1117, Amsterdam, The Netherlands

^4^ Bioimaging, In Vitro / In Vivo Translation (IVIVT), GlaxoSmithKline, Stevenage, United Kingdom

^5^ RadboudUMC, Department of Medical Oncology, Geert Grooteplein zuid 10, Nijmegen, The Netherlands

^6^ Amsterdam UMC location Vrije Universiteit Amsterdam, Department of Medical Oncology, Boelelaan 1117, Amsterdam, The Netherlands

**First and corresponding author:**

Name: Jessica E. Wijngaarden

E-mail: [j.e.wijngaarden@amsterdamumc.nl](mailto:j.e.wijngaarden@amsterdamumc.nl)

Address: De Boelelaan 1117, 1081 HV Amsterdam, The Netherlands

Telephone number: 020-4442863

Fax number: 020-4444329

**SUPPLEMENTAL DATA**


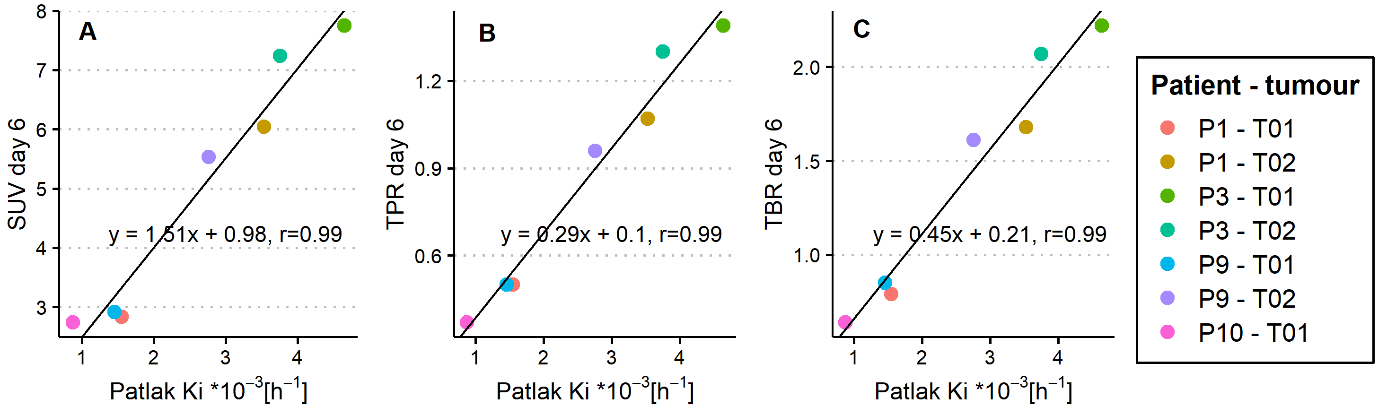


Supplemental Figure S1: Correlations between SUV (**A**) , TPR (**B**) and TBR (**C**) on day 6 and Patlak K_i_ values for ^89^Zr-anti-EGFR uptake in seven tumours of four patients.


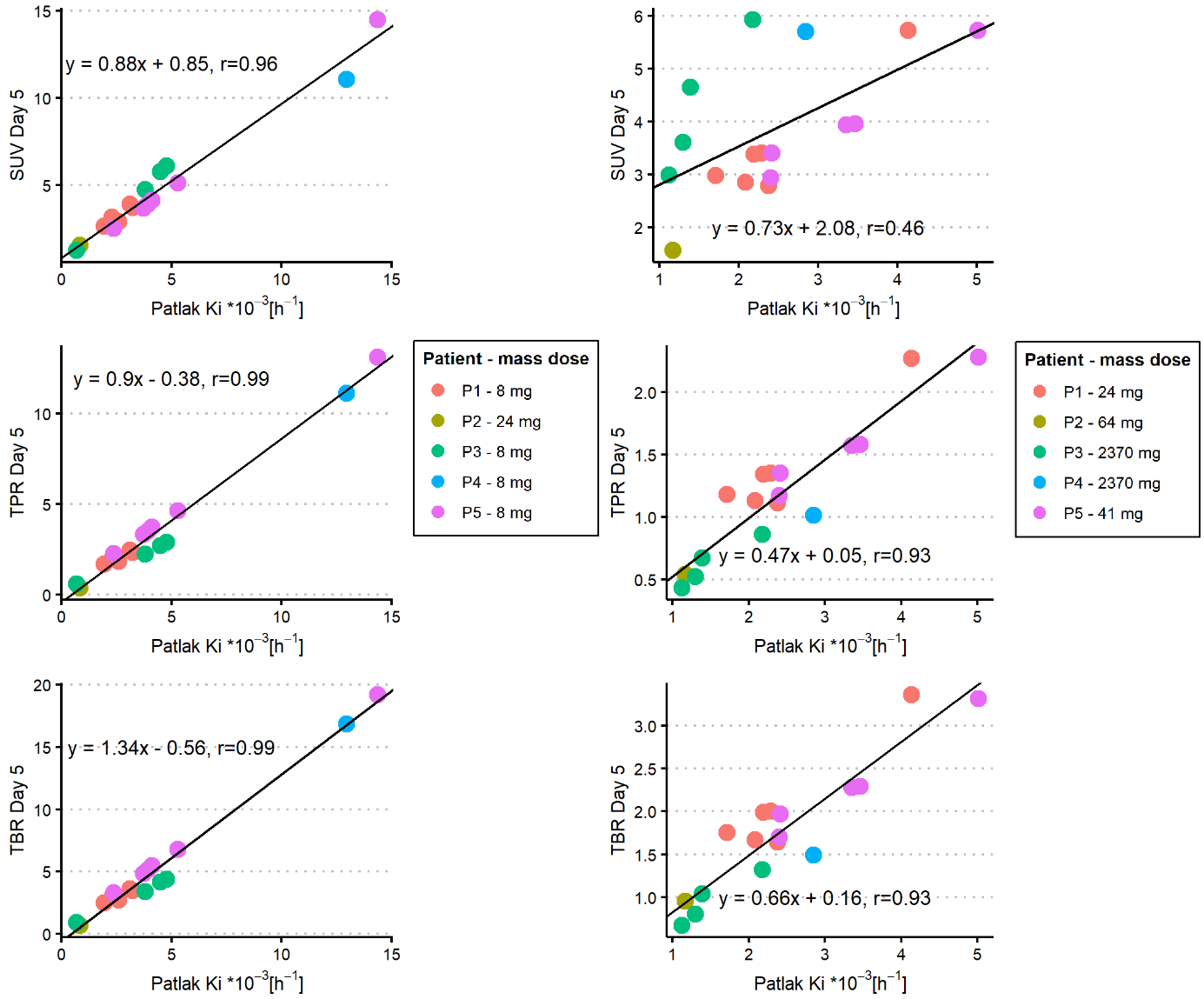


Supplemental Figure S2: Correlations between SUV, TPR and TBR on day 5 and Patlak K_i_ values for ^89^Zr-anti-HER3 uptake in tumours, for the first administration with similar mass doses (**A**&**C&E)** and the second administration with variable mass doses (**B**&**D&F**).
